# Satisfaction With Primary Care Among the Foreign-Born and the General Population in Finland: A Survey-Based study

**DOI:** 10.1177/00469580241252567

**Published:** 2024-05-06

**Authors:** Valentina Kieseppä, Regina García Velázquez, Tuulikki Vehko, Anu Castaneda, Hannamaria Kuusio

**Affiliations:** 1 Finnish Institute for Health and Welfare, Public Health and Welfare, Helsinki, Finland; 2 University of Oulu, Faculty of Medicine, Research Unit of Clinical Medicine, Oulu, Finland; 3 University of Edinburgh, Centre for Clinical Brain Sciences, Edinburgh, UK

**Keywords:** emigrants and immigrants, treatment satisfaction, primary health care, survey

## Abstract

Foreign-born people have been found to be less satisfied with health care than native populations across countries. However, studies on differences in satisfaction with treatment between different foreign-born groups are lacking. This study explores differences in satisfaction with primary health care between the foreign-born population from different regions of origins and the general population of Finland. The study uses survey data on foreign-born population (n = 2708) and general population (n = 6671) living in Finland who report using health services. Satisfaction with experienced respect for privacy during treatment, benefit of treatment and smoothness of treatment are predicted by region of origin using logistic regression. Almost all foreign-born groups were less likely to consider treatment appointments beneficial as compared to the general population. Some foreign-born groups (people from Southeast Asia and South and Central Asia) were more satisfied with smoothness of care compared to general population. People from East Asia were less likely than the general population to consider that their privacy had been respected during the examinations and treatment. While we made the positive finding of high overall satisfaction with treatment, we also found important differences between groups. In particular, appointments were found less useful among the foreign-born population. Perceived unusefulness of treatment might lead to underuse of health care, which might result in accumulation of untreated health problems. The results point toward potential development points in the health care system. Addressing these issues might help decrease health disparities between population groups.

**What do we already know about this topic?**
Across countries and health care providers, it has been consistently found that immigrants are less satisfied with health care compared to general populations.
**How does your research contribute to the field?**
Very little is known about how different immigrant groups differ from each other in terms of satisfaction with primary health care (i.e., how cultural background specifically affects the perceived treatment experience). To this research gap, our study provides insights.
**What are your research’s implications toward theory, practice, or policy?**
Our results point toward potential development points in the health care system. We found differences in patterns of satisfaction between different immigrant groups, and in particular, that most immigrant groups considered treatment less beneficial in comparison to the general population.

## Background

At the end of 2022, there were around 477 000 people born outside of Finland living in Finland (9% of the total population), an 8% increase from the previous year.^1^ The largest foreign-born groups in Finland are people born in the former Soviet Union or Russia, Estonia, Sweden, Iraq, China, and Somalia. As cultural diversity increases, it is important that the health care system evolves to be better equipped to provide treatment regardless of patients’ cultural background.

It has been repeatedly found that immigrants are less satisfied with health care,^2-5^ and find it less accessible^6^ compared to general populations. Immigrants assess interactions with physicians more negatively and rate the experience of continuity of treatment worse,^3^ and have been shown to be less satisfied with maternity care,^5^ rehabilitation care,^2^ and emergency care.^4^ In Finland, it has been shown that the foreign-born population experiences as much need for health services as the general population, but uses services less.^7^ Results of our previous study show that the foreign-born population, particularly those from Middle East and Africa, find treatment less accessible than others.^8^

The pattern of lower satisfaction among immigrants has been linked to, for example, linguistic and cultural barriers they might face in the health care system.^9-11^ In general, satisfaction with health care is positively associated with adherence to medication^12^ and favorable health outcomes,^13,14^ and thus such patterns of dissatisfaction among certain population groups might have important implications for population health.

Only few studies have investigated differences in satisfaction between groups of different regions of origins. In Norway, it has been shown that particularly people from Turkey, Iran, Pakistan and Vietnam are less satisfied with primary care than native Norwegians,^15^ and in Denmark, people from the Middle East have been found to be less satisfied with emergency care as compared to native Danes.^4^

Finland has a universal health care system which covers all residents with a residence permit and a permanent residence. There are three different health care systems: public health care, occupational health care (as an additional medical treatment provider), and private health care. These services are supplemented by third sector services (i.e., non-governmental non-profit organizations providing health care services) that provide, for example, peer-support and patient information in different languages. There are differences in user fees, scope and waiting times between the systems. Public health care is relatively affordable but often criticized for long waiting times for everyone.^16^ Employers are obligated to provide preventive health care services for their employees, but the scope of other occupational services varies.^17^ There are also private health services for those who have the possibility and preference to pay for their health services themselves (people with health insurance use private services more). Complicated health problems are usually treated within public health care.

Assessing satisfaction with health care among groups with different cultural backgrounds is important to gain information on the potential development points in the health care system. The aims of this study are to explore differences in satisfaction with primary health care visits to doctors and nurses between the foreign-born population from different regions of origins and the general population of Finland. We decided to study the satisfaction with primary care as the immigrant population of Finland are known to be particularly likely to use primary care services when using health care.^7^ The following aspects of satisfaction are explored: (1) the perceived respect for privacy during the treatment, (2) the perceived benefit of the treatment, and (3) the experience of smoothness of the treatment in terms of how smoothly the problem was handled and how well information was transferred between professionals.

## Methods

This study uses data from two surveys implemented by the Finnish Institute for Health and Welfare (THL): the Survey on Well-Being among Foreign Born Population (FinMonik) and the National FinSote Survey (FinSote) 2018. Informed consent was obtained from all participants involved in the surveys.

### The Foreign-Born Sample

The study participants were identified from the population register in March 2018. The survey sample was based on stratified random sampling, which is described in more detail elsewhere.^18^ The inclusion criteria were: 1) not born in Finland, 2) both parents or the only known parent not born in Finland, 3) must have lived in Finland for at least a year at the time of the sampling, 4) aged 18 to 64 at the time of sampling, and 5) did not move to Finland through adoption.

The data were collected between March 2018 and January 2019 primarily with an electronic questionnaire, which was supplemented with a paper questionnaire and telephone interviews. The invitation letter and the questionnaire were translated into 17 languages (from Finnish to Albanian, Arabic, Dari, Farsi, English, Spanish, Mandarin Chinese, Kurdish (Sorani), Polish, French, Swedish, Somali, Thai, Turkish, Russian, Vietnamese, and Estonian). The final sample consisted of 12 877 individuals, of whom 6836 responded. We included individuals who had completed the full version of the questionnaire and who were aged 20 or older (n = 6 312). The full methods of the FinMonik survey are described in more detail elsewhere.^18^

### The General Population Sample

The FinSote survey is an annual national survey on health, well-being and service use of the general population living in Finland. The data from the FinSote survey of 2018 are used as comparison data here.

The respondents selected were permanently living in Finland and over 20 years old. The questionnaire was sent to 60 000 individuals, of whom 26 422 individuals responded (response rate 45.3%). For the current study, individuals aged over 65 years were removed so that the samples would be comparable (n = 11 378, response rate 34.4%). As the respondents of the FinSote survey are randomly selected from the population register, the data includes a small number of foreign-born individuals, reflecting realistically the true Finnish general population.

### Visits to Doctors and Nurses in Primary Care

The respondents were asked to report separately the number of visits to doctors and/or nurses during the past 12 months at health centers, private medical clinics and/or in occupational health care due to illness, pregnancy or childbirth (visits at hospital outpatient clinics, dental appointments, or visits in third sector services were excluded). Only the individuals who reported at least one visit to either a doctor or a nurse in at least one of these health facilities were included in the study (foreign-born population n = 2708, general population n = 6671).

### Satisfaction Measures

The participants were asked to think about their experiences of using health services during the past 12 months and to evaluate how the following aspects were achieved in their case: *1) my privacy was respected in the examinations and treatment, 2) the treatment appointment was beneficial for me*, and *3) my problem was handled smoothly and information was transferred between professionals*. Response options were: *1) always, 2) most of the time, 3) sometimes, 4) never, 5) does not apply to me (I have not used health services).*

The three satisfaction items were dichotomized for the logistic regression models into categories *satisfied* (options *always* or *most of the time*) and *not-satisfied* (options *sometimes* or *never*), except for the privacy item. As the distribution of responses to the privacy item was strongly skewed toward the “always” option and very few individuals had chosen options “sometimes” or “never,” the same categorization could not be used as the estimates would not have been reliable. For this reason, the privacy item was dichotomized differently: *always*-*satisfied* (option *always*) and *not-satisfied* (options *most of the time*, *sometimes*, or *never*).

### Region of Origin

Information on the *region of origin* was obtained from the Digital and Population Services Agency and categorized as follows: 1) Russia, 2) Estonia, 3) Rest of Europe, North America and Oceania, 4) Middle East and North Africa, 5) Africa (excl. North Africa), 6) Southeast Asia, 7) East Asia, 8) South and Central Asia (incl. Bangladesh, Bhutan, India, Kazakhstan, Kyrgyzstan, Nepal, Pakistan, Tajikistan, Turkmenistan, Uzbekistan and Sri Lanka), 9) Latin America, 10) General population. The last category consists of the individuals from the FinSote survey data. The categorization was based on the United Nations Standard country or area codes for statistical use,^19^ with some modifications for the Finnish context. The “Russia” category also involves people whose country of origin is the former Soviet Union.

### Covariates

The variable *health care provider* was based on the reported health facility used and categorized as follows: *only public health care*, *only private health care*, *both public and private health care*. Occupational health care was categorized as private health care, as occupational health services are typically purchased from private providers. Information on *age* (categorized: 20–29, 30–49, and 50–64) and *sex* were obtained from the Digital and Population Services Agency. Information on *quality of life* and *chronic illness* were obtained from the questionnaires. Quality of life was measured with the item: *How would you rate your quality of life?* 1) *very poor*, 2) *poor*, 3) *neither poor nor good*, 4) *good*, 5) *very good*, and chronic illness was measured with the item: *Do you have a chronic illness or other chronic health problem*? 1) *yes* 2) *no*.

### Statistical Analysis

Differential item functioning (DIF) was measured to explore if the satisfaction items assessed the same latent characteristic for groups with different regions of origins by using the R’s *lordif* package.^20^ DIF was detected for the item *1) my privacy was respected in the examinations and treatment*. This should be taken into account when interpreting the results.

The two datasets were combined and transformed into a survey object (using R’s *survey* package^21^) using the appropriate survey weights (described in more detail in Kuusio et al^18^). The survey weights took into account the unequal sampling probabilities and the non-response. Chi-square tests of independence were used to study the differences in the variables of interest between the two samples. Separate logistic regression models were calculated for each outcome variable. The *region of origin* was the main explanatory variable in all the three models. Both unadjusted estimates and estimates adjusted with health care provider, sex, age, chronic illness and quality of life were produced. Nagelkerke *R*^2^ values were calculated as a measure of predictive power for each adjusted model. Participants with missing data on the variables of interest were excluded from the analyses.

## Results

There were significant differences between the foreign-born and the general population with respect to all descriptive variables except for sex. Individuals in the general population sample were older, reported their quality of life to be better, and had more often a chronic illness compared to the foreign-born population. Most participants reported using both public and private health care systems, but higher proportion of the general population reported using only private health care in comparison to the foreign-born population. The descriptive statistics of the sample are presented in Table 1.

**Table 1.** Descriptive Characteristics of Those Who Reported Visits to a Doctor or a Nurse at Primary Level in the Preceding 12-Month Period.

	Foreign-born	General population	*P*-value
	n (%)	n (%)	
Total	2708	6671	
Sex			.690
Female	1639 (54.0)	4142 (54.8)	
Male	1069 (46.0)	2529 (45.2)	
Age			**<.001**
20-29	563 (22.0)	959 (21.1)	
30-49	1586 (59.7)	2310 (44.7)	
50-64	559 (18.3)	3402 (34.2)	
Quality of life			**<.001**
Very poor	16 (1.2)	34 (0.7)	
Poor	65 (3.2)	232 (3.9)	
Neither poor nor good	479 (19.8)	1139 (15.6)	
Good	1581 (54.9)	3869 (57.0)	
Very good	509 (18.7)	1354 (22.1)	
Did not answer	58 (2.3)	43 (0.7)	
Chronic illness			**<.001**
Yes	1032 (37.2)	3465 (47.9)	
No	1646 (62.1)	3139 (51.4)	
Did not answer	30 (0.6)	67 (0.7)	
Health care use			**<.001**
Only public health care	878 (29.0)	1353 (19.4)	
Only private health care	630 (22.9)	2552 (39.9)	
Both public and private health care	1200 (48.1)	2766 (40.6)	
Region of origin			
Estonia	268 (13.2)		
Russia	826 (23.1)		
Rest of Europe, North America and Oceania	602 (22.8)		
Middle East and Northern Africa	331 (13.8)		
Africa (excl. North Africa)	137 (8.9)		
Southeast Asia	241 (6.7)		
East Asia	107 (3.6)		
South and Central Asia	122 (4.1)		
Latin America	74 (3.8)		

*Note*. FinMonik and FinSote studies (n, %) (absolute counts, sample-weighted percentages, *P*-values of chi-square tests). Bold values indicate significance level of *P* < 0.05.

The distributions of the responses to the satisfaction items are presented in Table 2. Trends in responses were similar between the samples and most participants were satisfied with their service experiences. Over half of the sample considered that their privacy had always been respected (66.2% of the foreign-born sample and 64.5% of the general population sample). Most participants considered that appointments were beneficial (over 70% of both samples), and problems were handled smoothly always or most of the time (also over 70% of both samples), although the foreign-born population were generally less satisfied with these aspects of care.

**Table 2.** Distributions of Responses to the Satisfaction Items Among the Foreign-Born and the General Population Samples (n, %) (Absolute Values, Sample-Weighted Percentages, *P*-Values of Chi-Square Tests).

	Privacy respected		*P*-value
	Foreign-born	General population	
Response	Crude N (%)	Crude N (%)	**<.001**
1. Always	1851 (66.2)	4216 (64.5)	
2. Most of the time	539 (21.9)	1640 (22.6)	
3. Sometimes	115 (5.4)	206 (3.4)	
4. Never	31 (0.9)	25 (0.4)	
5. Does not apply to me	132 (4.0)	517 (8.3)	
Unanswered	40 (1.6)	67 (0.8)	
	Appointment beneficial		
	Foreign-born	General population	
Response	Crude N (%)	Crude N (%)	**<.001**
1. Always	1129 (40.9)	2991 (44.3)	
2. Most of the time	927 (32.5)	2555 (37.7)	
3. Sometimes	447 (18.3)	545 (9.1)	
4. Never	65 (2.8)	57 (1.0)	
5. Does not apply to me	105 (3.5)	461 (7.2)	
Unanswered	35 (2.0)	62 (0.7)	
	Problem handled smoothly		
	Foreign-born	General population	
Response	Crude N (%)	Crude N (%)	**<.001**
1. Always	1119 (42.1)	2471 (37.3)	
2. Most of the time	914 (32.6)	2610 (37.9)	
3. Sometimes	419 (16.2)	864 (13.5)	
4. Never	82 (3.2)	135 (2.5)	
5. Does not apply to me	125 (3.8)	518 (7.9)	
Unanswered	49 (2,2)	73 (0.9)	

Note. Bold values indicate significance level of *P* < 0.05.

The results of the unadjusted and the adjusted logistic regression models are presented in Table 3.

**Table 3.** Satisfaction With Treatment as Predicted by Region of Origin (Ref. General Population) (Odds Ratios, 95% Confidence Intervals).

	Privacy		Beneficial		Problem handled smoothly	
	Unadjusted	Adjusted	Unadjusted	Adjusted	Unadjusted	Adjusted
	Odds ratios (95% CI)					
Russia and former Soviet Union	1.15 (0.86-1.54)	1.22 (0.88-1.69)	0.55***(0.39-0.76)	0.56** (0.39-0.80)	0.76 (0.57-1.02)	0.78 (0.58-1.05)
Estonia	0.97 (0.65-1.45)	1.08 (0.71-1.65)	0.33*** (0.21-0.50)	0.34***(0.22-0.54)	0.80 (0.50-1.27)	0.89 (0.55-1.44)
Rest of Europe, North America and Oceania	1.03 (0.75-1.42)	1.16 (0.84-1.60)	0.38***(0.26-0.54)	0.38***(0.26-0.55)	0.77 (0.53-1.12)	0.92 (0.64-1.33)
Middle East and North Africa	0.89 (0.56-1.40)	1.18 (0.73-1.90)	0.21***(0.13-0.32)	0.28***(0.17-0.45)	0.45***(0.28-0.71)	0.64 (0.39-1.06)
Africa (excl. North Africa)	0.97 (0.54-1.77)	0.93 (0.48-1.80)	0.45* (0.24-0.91)	0.47* (0.24-0.91)	1.06 (0.52-2.15)	1.14 (0.59-2.22)
Southeast Asia	0.59* (0.37-0.94)	0.66 (0.42-1.04)	1.36 (0.76-2.43)	1.53 (0.85-2.72)	1.92* (1.12-3.29)	2.27** (1.30-3.95)
East Asia	0.40** (0.21-0.75)	0.44* (0.22-0.89)	1.51 (0.64-3.50)	1.60 (0.68-3.76)	1.89 (0.87-4.09)	2.09 (0.91-4.78)
South and Central Asia	0.84 (0.45-1.55)	0.80 (0.41-1.57)	0.79 (0.40-1.57)	1.02 (0.45-2.34)	2.54* (1.14-5.64)	3.13* (1.26-7.78)
Latin America	2.09 (0.80-5.47)	2.30 (0.87-6.12)	1.04 (0.39-2.76)	0.90 (0.32-2.57)	1.38 (0.54-3.54)	1.48 (0.58-3.81)
	Nagelkerke *R*^2^					
		0.08		0.14		0.11

* *P* < .05. **P < .01. ***P < .001.

Our most important finding was that both in the unadjusted and in the adjusted models, respondents from Russia, Estonia, Rest of Europe, North America and Oceania, Middle East and North Africa and Africa (excl. North Africa) had lower odds for considering treatment beneficial as compared to the general population.

In the unadjusted model, individuals from Southeast Asia and East Asia had lower odds for considering that their privacy had always been respected compared to the general population. In the adjusted model, only individuals from East Asia were significantly less likely to respond that their privacy had always been respected as compared to the general population. Neither in the adjusted nor in the unadjusted model was any group significantly more likely to consider that their privacy had always been respected in comparison to the general population.

In the unadjusted model, respondents from Southeast Asia and South and Central Asia were more likely to consider that their problem had been handled smoothly in comparison to the general population. The estimates remained similar in the adjusted model. No group was significantly less likely to be satisfied with the smoothness of treatment in comparison to the general population; however, in the unadjusted model, individuals from the Middle East and North Africa were significantly less likely than the general population to consider that their problem had been handled smoothly.

## Discussion

We found that both the foreign-born population and the general population were generally satisfied with the treatment they received. However, there were important differences in satisfaction between groups. In particular, almost all foreign-born groups found treatment less beneficial than the general population.

One of the major reasons for dissatisfaction with health care among immigrants are problems in communication,^11^ which can decrease the perceived usefulness of the appointment significantly. A study found that immigrants report worse outcomes after care^22^ and there is also evidence that treatment for immigrants might be less effective when assessed by objective measures.^23^ Different expectations of treatment due to different health care systems across countries might also affect the perceived usefulness of treatment.^11^ Overall, not finding treatment beneficial is a significant problem regardless of the objective benefit: if health care is not deemed useful, individuals are less likely to use health care in the future, which in turn might lead to accumulation of untreated health problems.

We also found that respondents from East Asia and Southeast Asia were less likely than the general population to find that their privacy had always been respected during the treatment. This might relate to cultural differences in expectations of care. For example, a study on cervical cancer screenings among Chinese American women found that many of the participants believed “feminine problems” should not be shared,^24^ and a study on Southeast Asian doctor-patient communication style highlights the importance of maintaining harmony and politeness during the visit.^25^ However, it should be noted that the item measuring privacy concerns was the one where we detected differential item functioning, which implies that the item seems to have meant something else for the foreign-born as compared to the general population. In addition to this, as the privacy item was dichotomized differently than the other two items, interpretation of these results should be done carefully.

People from Southeast Asia and South and Central Asia were more satisfied with smoothness of care compared to the general population. There were no other statistically significant differences in the experience of smoothness of care between groups. Overall, this result seems positive as it points toward no major effects of cultural background to the perception of smoothness of health care. Experiences of health care use in the country of origin might also affect this perception, as health care in the country of origin is typically compared to the health care in the host country.^11^

There are several mechanisms which might explain the patterns we found. Dissatisfaction with health care among immigrant patients is probably most often linked to communicational problems due to both language and cultural barriers.^5,9-11,26,27^ As communicational issues can occur even when there is a shared language,^28^ and the patients’ and health care professionals’ experiences of treatment might differ from each other even among general populations,^29^ the issue of communication is particularly important among immigrants. In addition, navigating health care systems requires health literacy—for example, knowledge on how to describe symptoms to health care professionals or the ability to understand what your doctor is saying to you.^30^ Studies have shown that immigrants have lower health literacy compared to native populations.^31^ Lower health literacy among immigrants might also explain some of the differences in satisfaction found in our study.

Cultural insensitivity or lack of cultural knowledge among the health care professionals is another typically reported problem.^9-11^ Unfortunately, experiences of discrimination by health care staff are also not uncommonly reported.^5,9,10,26,28^ Medical mistrust has also been associated with lower satisfaction with care.^32^ In addition, there might be differences in referrals to treatment between different groups.^11^ Some foreign-born groups have been shown to be underrepresented in mental health care services in Finland.^33,34^

There were also significant differences in sociodemographic characteristics between the samples: individuals from the general population were, on average, older, reported higher quality of life and reported having a chronic illness more often than the foreign-born population. Most of the participants reported visits to both public and private health care. This is typical for the Finnish health care system, as the system naturally results in using multiple health service providers. A significantly higher proportion of the general population had used only private health care in comparison to the foreign-born population. Results with similar trends have been obtained from an earlier Finnish study.^35^ As the models were adjusted for these sociodemographic variables, the observed differences in satisfaction with treatment were independent from them. Still, it is worth noting that there are significant differences in the providers of services used by different population groups, which should be taken into account when planning the services.

Unfortunately, we did not have information on employment, education or other measures of socioeconomic status, even though immigrants are known to differ from the general population with regard to these aspects. In Finland, while the overall education level among the immigrant population is estimated to be quite high, there are differences in education level between groups, employment rates are lower among the foreign-born population, and many immigrants report being overqualified to the employment they have received in Finland.^7^ As socioeconomic status is related to both need for health care and health care utilization,^36^ it is important to take into account this diversity when planning the services.

Overall the patterns we found are worrying, as perceived unusefulness of treatment might lead to not using health care in the future, which might result in accumulation of untreated health problems among certain groups. These results emphasize the need of the health care system to be culturally sensitive and inclusive in order to reduce health inequalities between population groups and to provide appropriate care for people of all backgrounds. Potential development points could be, for example, ensuring the availability of interpreters and providing cultural sensitivity training for health care professionals. In addition, the current national emphasis on digitalization of health care and social welfare^37^ can create important new possibilities and challenges for immigrants: while engaging in digital services might provide financial and time benefits, technological and language difficulties might create the biggest barriers for those in the most vulnerable position.^38^

### Limitations

There are some limitations associated with this study. There were some important variables that we could not control for: namely, region of residence, socioeconomic status (although health care provider gives some indication of this) and length of stay. In Finland, there are regional differences in access to primary health care. While in rural areas there are more general practitioners per resident compared to urban areas, distances to services are greater.^39^ Regional differences might explain some of the disparities between the two populations, as the foreign-born population are more concentrated on the metropolitan area, but the direction of this potential influence is not straightforward. Regarding the effect of length of stay, people who have lived in the host country longer are more likely to be more familiar with the culture, health care system and language, thus lowering the amount of potential cultural and linguistic barriers they might experience. Some of the observed differences might have been explained by differences in patterns of length of stay among different immigrant groups.

The possibility of non-response bias cannot be ruled out, although we have attempted to minimize it with sample weights. Still, it is possible that the individuals who did not respond to the survey are significantly different in their patterns of health care experiences and satisfaction.

Finally, because of the differences of health care systems across countries, generalization of the results should be done carefully.

## Conclusions

Overall, our study made the positive finding that both the foreign-born and the general population were satisfied with the treatment they had received in primary health care: most participants considered that their privacy had been respected, the appointments had been beneficial, and their problems had been handled smoothly always or most of the time. However, most foreign-born groups were more likely to consider that the treatment had not been beneficial compared to the general population. These results were independent of the provider of health service. The disparity might be attributable to, for example, communicational issues, cultural insensitivity of the health care system, or different expectations of health care. A lack of perceived benefit from treatment might lead to decreased health care use in the future, and thus accumulated health problems in certain population groups. Therefore, these results point toward potential targets for development in the health care system.
